# The palmar metric: A novel radiographic assessment of the equine distal phalanx

**Published:** 2014-08-09

**Authors:** M.A. Burd, J.J. Craig, M.F. Craig

**Affiliations:** 1 *Animal Science Department, California Polytechnic State University, One Grand Avenue, San Luis Obispo, California 93401, USA*; 2 *Eponatech, Inc., P.O. Box 361, Creston, CA 93432, USA*

**Keywords:** Horse, Metric, Palmar, Phalanx, Radiograph

## Abstract

Digital radiographs are often used to subjectively assess the equine digit. Recently, quantitative and objective radiographic measurements have been reported that give new insight into the form and function of the equine digit. We investigated a radio-dense curvilinear profile along the distal phalanx on lateral radiographs we term the Palmar Curve (PC) that we believe provides a measurement of the concavity of the distal phalanx of the horse. A second quantitative measurement, the Palmar Metric (PM) was defined as the percent area under the PC. We correlated the PM and age from 544 radiographs of the distal phalanx from the left and right front feet of various breed horses of known age, and 278 radiographs of the front feet of Quarter Horses. The PM was negatively correlated with age and decreased at a rate of 0.28 % per year for horses of various breeds and 0.33 % per year for Quarter Horses. Therefore, veterinarians should be aware of age related change in the concave, parietal solar aspect of the distal phalanx in the horse.

## Introduction

Radiographic assessment of the distal phalanx is the backbone of the veterinary evaluation of the equine digit. Knowledge of radiographic anatomy and methods to obtain optimal radiographs allow the veterinarian to render a subjective evaluation of the digit (Linford *et al.*, 1993; Redden, 2003; Turner, 2006). However, advances in digital radiology now support accurate, quantitative, measurements (Craig *et al.*, 2001; Rocha *et al.*, 2004; Kummer *et al.*, 2006).

While the elegant form and function of the distal phalanx have been described, including the distinct concave, parietal solar surface, little information exists regarding the manner in which this form may change as the horse ages (Parks, 2003).

Therefore, our objectives were to describe a quantitative measurement of the concave, parietal solar surface of the distal phalanx, the Palmar Metric (PM), and demonstrate the manner in which the PM changes with age.

## Materials and Methods

**Fig. 1:**
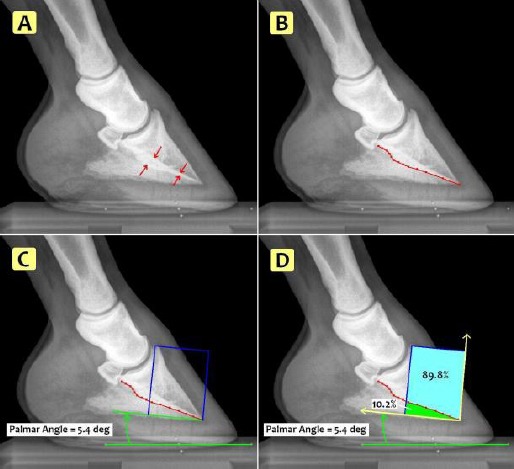
Determination of Palmar Curve (PC) and Palmar Metric (PM). **(A)** The radio-dense line along the concave, parietal solar surface of the distal phalanx on lateromedial (LM) radiograph indicated by red arrows. **(B)** The PC indicated by the red line on LM radiograph. **(C)** A coordinate system set at the tip of the distal phalanx using a line along the palmar angle and a vertical line through the extensor process. **(D)** The PM determined from the % area of the reference rectangle under the PC.

The Palmar Metric (PM) was developed using high quality, lateromedial (LM) digital radiographs of the distal phalanx of the horse. Using Metron-DVM (Metron-DVM Imaging Software, Eponatech, Inc., P.O. Box 361, Creston, CA 93432), a line termed the Palmar Curve (PC), was traced along the radio-dense line of the concave parietal solar surface of the distal phalanx from the tip of the distal phalanx along the most palmar aspect of the line to the proximal palmar articular surface (Fig. 1A and 1B). The PM was calculated by creating a coordinate system within the radiograph based on the PC and position of the distal phalanx (Fig. 1C). A reference rectangle was then constructed and the area within the rectangle defined above and below the PC. The PM was expressed as a percentage of the area under the PC relative to the area of the reference rectangle (Fig. 1D).

To determine the repeatability of the PM, we assessed intra-user and inter-user precision errors (SD) and coefficients of variation (CV) associated with this measurement (Glüer *et al.*, 1995). Each of four users measured the PM from 50 randomly chosen radiographs, repeating the measurement after a two-week time interval. Effect of radiographic position due to linear and up to 10 degrees of rotation from the standard lateral to medial position was assessed in the horizontal and vertical directions. Linear regression was used to assess changes in PM with radiographic positioning.

Subsequently, radiographs were obtained from participating equine veterinary clinics, and four independent users performed a retrospective analysis of the PM from 543 high quality LM digital radiographs of the front feet of horses of known age. Of these 543 radiographs, 219 left and right pairs were analyzed for differences in PM between left and right radiographs.

Differences in PM for left and right radiographs were analyzed using a two sample Student’s *t* test. Linear correlation was used to assess the relationship between PM and age for all radiographs. All statistical tests were performed using Minitab and were based on a 2-sided null hypothesis of no difference and a level of significance set at 0.05.

## Results

Depending on the user, precision errors (SD) ranged from 0.05 to 0.71, while the coefficients of variation (CV) ranged from 0.67 to 14.10 %. The overall PM precision error (SD) was 1.13 with a CV of 12.14 %. There was no statistical difference in determination of the PM associated with changes in radiographic positioning in the horizontal and vertical linear directions. Additionally, there was no difference in PM determination due to rotation up to 10 degrees from the standard lateromedial (LM) radiographic position.

**Fig. 2:**
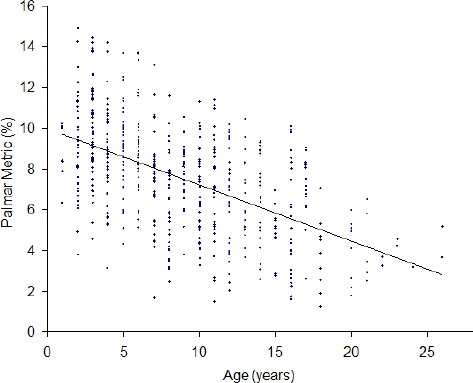
Palmar Metric (%) determined from 543 lateromedial radiographs of the distal phalanx of horse of known age. Graph includes linear regression line: y= -0.28x + 9.98, r^2^=0.28, *P*<0.05.

Mean PM ± SD and mean age ± SD from all radiographs in the study was 7.55 ± 2.70 % and 8.83 ± 5.13 years and mean PM ± SD are shown for each age class (Table 1). For each age class, there was no significant difference in mean PM between left and right radiogfiraphs. The slopes of the regression lines for the PM and age for left (-0.27 ± 0.03 % per year) and right (-0.29 ± 0.03 % per year) were not significantly different (*P*=0.75). The PM was negatively correlated with age and decreased by 0.28 ± 0.02% per year (Fig. 2, r^2^=0.28, *P*<0.05).

**Table 1:** Mean ± SD Palmar Metric (%) determined from 543 lateromedial radiographs of horses in each age class.

*Age (years)*	*N*	*Mean PM ± SD (%)*
1	7	8.76 ± 1.44
2	34	9.15 ± 2.56
3	54	9.94 ± 2.33
4	38	8.77 ± 2.40
5	40	9.10 ± 2.41
6	38	8.86 ± 2.10
7	43	7.42 ± 2.21
8	34	6.36 ± 2.37
9	29	7.78 ± 1.47
10	36	6.85 ± 2.09
11	34	7.04 ± 2.50
12	31	6.74 ± 2.45
13	21	6.57 ± 2.07
14	22	6.53 ± 2.13
15	11	4.93 ± 1.26
16	26	5.30 ± 2.75
17	16	7.14 ± 1.64
18	9	3.81 ± 1.82
20	8	4.01 ± 1.59
21	6	4.24 ± 1.62
22	2	3.46 ± 0.34
23	2	4.39 ± 0.26
26	2	4.42 ± 1.04

## Discussion

Radiographic assessment of the equine digit is important because valuable information can be obtained regarding the distal phalanx. While the radiographic appearance of the distal phalanx has been described, to our knowledge, the Palmar Metric (PM) is the only quantitative, radiographic measurement of the concave, parietal solar surface of the distal phalanx (Rendano and Grant, 1978). Analysis of precision errors (0.05-0.71) and CV (0.67-14.1 %) indicated there was a range of proficiency demonstrated by the individual users in determining the PM.

The overall precision error and CV for the PM indicated one can expect up to a 14% difference in the PM dependent on user expertise. The PM was not influenced by radiographic positioning, however, this was only determined for what we considered minor alterations from the standard lateromedial (LM) positioning. This indicated for an experienced user with radiographic positioning artifact less than 10 degrees, the PM can be reliably determined.

Because the PM is a ratio, it is not dependent on size of the distal phalanx and can be assessed independent of breed related differences in foot size. The PM is dependent on the shape of the concave, parietal solar surface of the distal phalanx and, as the concavity of the solar surface increases, the PM increases. In our study, the PM ranged from 1.24 - 16.74% and was shown to be negatively correlated with age. Therefore, young horses had a greater PM than older horses in this study.

In humans, the age-related, gradual demineralization of bones and concomitant change in bone shape is a well-studied phenomenon (Lindsay, 1995; Maggio *et al.*, 1997). To our knowledge, this has not been reported for the distal phalanx of the horse. We surmise the gradual demineralization of the distal phalanx, especially along the distal solar margin, decreases the solar concavity over time.

The distal phalanx of the horse changes dramatically in form in response to disease (Watters *et al.*, 1978). While the PM for left and right radiographs was not significantly different at any age class in our horses, we suspect unilateral foot pathology would result in differences between the left and right PM for an individual horse. Thus, differences in the PM between the left and right forelimbs may offer insight into the pre-existence, severity, and chronic nature of disease. Additionally, geriatric horses experience greater musculoskeletal disease than younger horses, which may influence the PM (Hunt 2002; Brosnahan and Paradis, 2003).

Therefore, the influence of digital pathology on the PM requires further investigation. From a clinical perspective, veterinarians should be aware of the age related change of the concave, parietal solar surface of the distal phalanx. It is hoped that a better understanding of this change throughout the lifetime of the horse, quantified by the PM, will yield improvement in hoof care and treatment of foot related disease.
